# Quantitative PCR Method for Enumeration of Cells of Cryptic Species of the Toxic Marine Dinoflagellate *Ostreopsis* spp. in Coastal Waters of Japan

**DOI:** 10.1371/journal.pone.0057627

**Published:** 2013-03-13

**Authors:** Naohito Hariganeya, Yuko Tanimoto, Haruo Yamaguchi, Tomohiro Nishimura, Wittaya Tawong, Hiroshi Sakanari, Takamichi Yoshimatsu, Shinya Sato, Christina M. Preston, Masao Adachi

**Affiliations:** 1 Kochi University, Monobe, Nankoku, Kochi, Japan; 2 The United Graduate School of Agricultural Sciences, Ehime University, Matsuyama, Ehime, Japan; 3 Royal Botanic Garden Edinburgh, Edinburgh, United Kingdom; 4 Monterey Bay Aquarium Research Institute, Moss Landing, California, United States of America; University of New South Wales, Australia

## Abstract

Monitoring of harmful algal bloom (HAB) species in coastal waters is important for assessment of environmental impacts associated with HABs. Co-occurrence of multiple cryptic species such as toxic dinoflagellate *Ostreopsis* species make reliable microscopic identification difficult, so the employment of molecular tools is often necessary. Here we developed new qPCR method by which cells of cryptic species can be enumerated based on actual gene number of target species. The qPCR assay targets the LSU rDNA of *Ostreopsis* spp. from Japan. First, we constructed standard curves with a linearized plasmid containing the target rDNA. We then determined the number of rDNA copies per cell of target species from a single cell isolated from environmental samples using the qPCR assay. Differences in the DNA recovery efficiency was calculated by adding exogenous plasmid to a portion of the sample lysate before and after DNA extraction followed by qPCR. Then, the number of cells of each species was calculated by division of the total number of rDNA copies of each species in the samples by the number of rDNA copies per cell. To test our procedure, we determined the total number of rDNA copies using environmental samples containing no target cells but spiked with cultured cells of several species of *Ostreopsis*. The numbers estimated by the qPCR method closely approximated total numbers of cells added. Finally, the numbers of cells of target species in environmental samples containing cryptic species were enumerated by the qPCR method and the total numbers also closely approximated the microscopy cell counts. We developed a qPCR method that provides accurate enumeration of each cryptic species in environments. This method is expected to be a powerful tool for monitoring the various HAB species that occur as cryptic species in coastal waters.

## Introduction

The occurrence of harmful algal blooms (HABs) is a significant and increasing threat to human health as well as fishery resources on a global scale [1], [2], [3]. Monitoring coastal waters for the presence of HAB species is essential for assessing the risk of bloom formation and for mitigating impacts of blooms that may affect aquaculture sites or other areas of concern to public health and wildlife. Because of the complexity, scale, and transient nature of HABs, their monitoring and prediction can be enhanced with rapid and real-time observations. These requirements cannot always be met with traditional approaches that depend on microscopic examination for identifying problem species. Lack of taxonomic capability required for species identification can negatively impact efforts to provide “early warning” bloom monitoring. An alternative approach is to use molecular methods for detection and as a proxy to estimate HAB cell numbers [4]. Such methods are especially well suited for detecting species that are not easy to identify using standard microscopy techniques.

Recently, molecular analytical methods such as quantitative PCR (qPCR) [5] have been applied for detection of various species of microalgae. For HAB species, qPCR methods have been developed for a variety of *Alexandrium* [6], [7], [8], [9], *Aureococcus* [10], *Chattonella* [11], [12], [13], [14], [15], *Heterosigma* [11], [12], [13], [14], [15], *Gambierdiscus* [*16*],*Karenia* [17], *Lingulodinium* [18], *Ostreopsis* [*19*] and *Pfiesteria* [18], [20], [21], [22], [23], [24], [25] species. The cell quantification methods by qPCR are broadly classified as “relative” and “absolute” methods [26].

“Relative” qPCR measures the differences in “relative” abundances of cells in the target samples and reference samples without determining the actual copy numbers of the target DNA. “Relative” qPCR methods have been applied for detection and quantification of various HAB species [6], [7], [8], [9], [11], [12], [13], [14], [15], [16], [17], [18], [19], [20], [21], [22], [25]. In all studies except for the two studies described below, cultured cells have been used as reference samples. While this method is relatively straightforward, the application of qPCR to environmental studies sometimes produces variable results [27], [28], [29] because the amplification efficiencies and DNA recovery efficiencies of plasmids or the laboratory cultures used for generation of a standard curve may not accurately represent the amplification efficiencies and recovery efficiencies of DNA extracted from environmental samples. In order to overcome this problem, the comparative cycle threshold (Ct) method [30] has been applied to harmful microalgal cell quantification using natural environmental samples as reference samples [12]. In this method, genomic DNA extracted from environmental samples containing target cells that had been enumerated under a microscope, were used as templates in qPCR and represent reference samples ( = Calibrator samples). Cell counts and Ct values of target species in the calibrator samples provided a means for calculating cell abundances in other environmental samples based on the assumption that the amplification efficiency of the environmental sample closely approximated that of the calibrator sample [12]. Another approach also utilizes a relative qPCR method for enumeration of a HAB species in marine environment and environmental samples as a reference [19]. The method relies on a ‘gold standard’ created with pooled crude extracts from environmental samples including cells of the target species that had been enumerated by microscopic examination. The two molecular approaches are applicable for the monitoring of target harmful species that can be identified by their morphological characteristics. For samples containing “cryptic” species, preparation of a “calibrator” or “gold standard” sample is difficult because they cannot be identified based on their morphological features.

In contrast, the “absolute” qPCR method is thought to have the potential for enumerating cryptic species as the method does not rely on a reference culture or sample to generate the standard curve. “Absolute” qPCR utilizes standard curves that are generated by amplifying a dilution series of plasmids containing the target gene sequence and requires knowledge of the gene copy number per cell of target cryptic species. The gene copy number per cell of target cryptic species is determined by qPCR using single cells isolated from environmental samples as the template. Then, the cell numbers of target species in environmental samples are obtained by division of the total numbers of target genes in a sample by the number of target genes per cell. Galluzzi et al. applied an “absolute” qPCR method considering the number of target genes per cultured cell for detection and quantification of the HAB species, *Alexandrium minutum* [7]. While the principle and process of the “absolute” qPCR method are relatively straightforward, it was reported that the method had low precision and repeatedly overestimated the number of cells of the target species [31]. This is partly due to the general variability in gene copy number between different strains of the target species and the growth phase of each culture [7], [19].

Here, we present a new approach using the “absolute” qPCR method that is applicable for detection and enumeration of cryptic species. The dinoflagellate *Ostreopsis* spp. was chosen as model HAB species for this investigation. Species of this genus are distributed from tropical to temperate coastal areas and are associated with the production of potent palytoxin-like compounds [32], [33]. In recent years, HABs by *Ostreopsis* species have occurred frequently in coastal waters throughout the world and may have a negative impact on environmental quality and human health [34], [35], [36], [37]. In Japanese coastal waters, *Ostreopsis* species have been recorded since the late 1970s and palytoxin-like compounds were found in the cells of the species [38], [39], [40], [41], [42], [43]. Recently, Sato et al. reported the phylogeography of the *Ostreopsis* species along the West Pacific coast and revealed that there are four major *Ostreopsis* species: *O*. cf. *ovata*, *Ostreopsis* sp. 1, *Ostreopsis* sp. 5 and *Ostreopsis* sp. 6 [44]. Toxicities of the species are considerably different from each other. For example, *Ostreopsis* sp. 5 is non-toxic and *Ostreopsis* sp. 1 is strongly toxic [44]. Of the four species, *O*. cf. *ovata* and *Ostreopsis* sp. 1 are cryptic species of *O*. *ovata*.

Our “absolute” method, targeting the cryptic species of *Ostreopsis,* accounts for the efficiency of DNA extraction and recovery, qPCR amplification efficiency, and the number of copies of the ribosomal RNA gene (rDNA) per cell of each target cryptic species in environmental samples. Accuracy of this method was evaluated using environmental samples spiked with known numbers of cultured cells of cryptic species. In addition, we also applied this method to enumerate cryptic HAB species in environmental samples. Application of this method allows monitoring target cryptic HAB species in marine ecosystems.

## Materials and Methods

### Ethics Statement

No specific permits were required for the described field studies. No specific permission was required for any locations and activities. The locations are not privately owned or protected in any way. No activities during the field study involved any endangered or protected species.

### Culture Samples

Strains of the various species of *Ostreopsis* [44], *Gambierdiscus* spp. and *Coolia* sp. isolated from Japanese coastal waters and used in this study are listed in Table 1. All strains were cultured in f/2 medium with a 12 h light and 12 h dark cycles (white light at 100 µmol photons m^−2^ s^−1^) illumination at 25°C.

**Table 1:** Culture sample list.

Species	Clade*	Strain name	Locality
*Ostreopsis* cf. *ovata*	A	s0579	Kubazaki, Kohama Island, Okinawa, Japan
*Ostreopsis* cf. *ovata*	A	s0619	Nagata cho, Nagasaki city, Nagasaki, Japan
*Ostreopsis* cf. *ovata*	A	s0662	Tei, Konan City, Kochi, Japan
*Ostreopsis* cf. *ovata*	A	s0667	Tei, Konan City, Kochi, Japan
*Ostreopsis* cf. *ovata*	A	s0711	Nishidomari, Otsuki Town, Kochi, Japan
*Ostreopsis* cf. *ovata*	A	s0713	Nishidomari, Otsuki Town, Kochi, Japan
*Ostreopsis* cf. *ovata*	A	s0731	Nishidomari, Otsuki Town, Kochi, Japan
*Ostreopsis* cf. *ovata*	A	KAC85	La Spezia beach, Genoa, Ligurian Sea, Italy
*Ostreopsis* sp. 1	B-1	s0618	Nagata cho, Nagasaki city, Nagasaki, Japan
*Ostreopsis* sp. 1	B-1	s0659	Tei, Konan City, Kochi, Japan
*Ostreopsis* sp. 1	B-1	s0716	Nishidomari, Otsuki Town, Kochi, Japan
*Ostreopsis* sp. 1	B-1	s0737	Nishidomari, Otsuki Town, Kochi, Japan
*Ostreopsis* sp. 1	B-1	s0772	Bishagoiwa, Muroto City, Kochi, Japan
*Ostreopsis* sp. 5	C-1	s0577	Haemida, Iriomote Island, Okinawa, Japan
*Ostreopsis* sp. 5	C-2	o07421-2	Bishagoiwa, Muroto City, Kochi, Japan
*Ostreopsis* sp. 6	D-2	s0587	Haemida, Iriomote Island, Okinawa, Japan
*Ostreopsis* sp. 6	D-2	ISC6	Kabira, Ishigaki Island, Okinawa, Japan
*Ostreopsis* sp. 6	D-2	ISC7	Kabira, Ishigaki Island, Okinawa, Japan
*Gambierdiscus* sp. type 1		KW070922_1	Kashiwajima, Ohtsuki Town, Kochi, Japan
*Gambierdiscus* sp. type 2		T070411_1	Tei, Konan City, Kochi, Japan
*Gambierdiscus australes*		I080606_1	Irabujima Island, Miyakojima City, Okinawa, Japan
*Coolia* sp.		S32C	Susaki Town, Kochi, Japan
*Coolia* sp.		S33C	Susaki Town, Kochi, Japan
*Coolia* sp.		S34C	Susaki Town, Kochi, Japan

* See Fig. 1 of Sato et al. (2011) [44].

### Quantitative PCR (qPCR Assay)

Primer and probe sites for *O*. cf. *ovata*, *Ostreopsis* sp. 1, *Ostreopsis* sp. 5 and *Ostreopsis* sp. 6 were identified by aligning the D8/D10 region of the 28S rDNA of the four species of *Ostreopsis* from Japanese coastal waters reported by Sato et al. [44] using ClustalW [45]. The primers and probes were designed utilizing the Primer Express software (Version 3.0, Applied Biosystems, Tokyo, Japan). Primer and probe sequences are shown in Table 2. The TaqMan probes used in this study (Table 2) were synthesized (Applied Biosystems) with a 6-FAM (6-carboxy fluorescein) reporter dye at the 5′ end and with MGB (Minor Groove Binding moiety) at the 3′ end.

**Table 2:** List of primer and probe sequences for Q-PCR of *Ostreopsis* species and for Q-PCR of pGEM plasmid.

Primer and probe set	DNA target	Primer or probe name	Sequence (5'-3')	Final primer or probe concentration
*O*. cf. *ovata* set	28S rDNA D8/D10 of *O.* cf. *ovata*	O. cf. ovata_Forward_2	GGCAAGTTGTTGCTTGACTGTAAA	400 nM
		O. cf. ovata_Reverse_2	AACTCCCCACCTAACTATGTTTTCC	400 nM
		O. cf. ovata_Probe_2	TGTAGTTTGTGCACTTGTG	250 nM
*Ostreopsis* sp.1 set	28S rDNA D8/D10 of *Ostreopsis* sp. 1	O. sp. 1_Forward_2	CTGTGGCAACTTGTTGTGTGAAT	200 nM
		O. sp. 1_Reverse_2	CCCCACCTAACTGTGTTTTCCA	200 nM
		O. sp. 1_Probe_2	CTGAAGTTTGTGCACTTGT	250 nM
*Ostreopsis* sp. 5 set	28S rDNA D8/D10 of *Ostreopsis* sp. 5	O. sp. 5_Forward_1	GTCCTTGCACTTGCCTGTTGT	200 nM
		O. sp. 5_Reverse_1	CCTGACGCAGTATTCCACACA	500 nM
		O. sp. 5_Probe_1	TTATAAGCCATTATGTATTGGTG	250 nM
*Ostreopsis* sp. 6 set	28S rDNA D8/D10 of *Ostreopsis* sp. 6	O. sp. 6_Forward_1	TGTTCTTGCATTTGCATGTTGTT	200 nM
		O. sp. 6_Reverse_1	CTGACACATTATTCCACACAAATACG	500 nM
		O. sp. 6_Probe_1	CTCTCCAGCCATTGTATGT	250 nM
pGEM set	pGEM-3Z plasmid	M13F	CCCAGTCACGACGTTGTAAAACG	900 nM
		pGEM R	TGTGTGGAATTGTGAGCGGA	900 nM
		pGEM Probe	CACTATAGAATACTCAAGCTTGCATGCCTGCA	250 nM

For detection and enumeration of pGEM-3Z (Promega, Tokyo, Japan), M13F primer and pGEM-R primer [25] were synthesized (FASMAC Co., Ltd., Kanagawa, Japan) and pGEM probe [25] was synthesized (Operon Inc., Tokyo, Japan) with a 6-FAM reporter dye at the 5′ end and BHQ (Black Hole Quencher) dye at the 3′ end. All qPCR assays were performed using Premix EX Taq™ (TaKaRa Bio, Shiga, Japan). Optimized 20ul reactions contained 1X Premix Ex Taq™ (TaKaRa Bio, Shiga, Japan), 1X ROX Reference Dye (TaKaRa Bio, Shiga, Japan), 2 µl of template DNA solution (see below), and primer and probe concentrations as listed in Table 2. All *Ostreopsis* and pGEM-3Z assays were performed with an ABI Prism 7300 SDS (Applied Biosystems, Tokyo, Japan) using the following quantification cycling protocol; 95°C for 30 sec. followed by 40 cycles at 95°C for 5 sec. and 60°C for 31 sec. All samples were analyzed in triplicate and the mean and standard deviation were calculated. No template controls (NTCs) using distilled water were conducted for each qPCR run to ensure that the results were not influenced by contamination. Acquisition of qPCR data and subsequent analysis were carried out using 7300 SDS Version 1.4 (Applied Biosystems, Tokyo, Japan). Fluorescence threshold was held at 1.0 and the threshold cycle (Ct) for each reaction was determined.

### Preparation of Plasmid Templates for qPCR

Partial 28S rDNA D8/D10 regions were amplified with F08 and RB primers [46] from strains of s0662 (*O.* cf. *ovata*), s0716 (*Ostreopsis* sp. 1), o07421-2 (*Ostreopsis* sp. 5) and s0587 (*Ostreopsis* sp. 6) according to the method described by Sato et al. [44]. PCR products were cloned into pMD20 plasmid vector (TaKaRa Bio, Shiga, Japan) following the manufacturer’s protocol. The plasmids were extracted using PureYield™ Plasmid Miniprep System (Promega, Tokyo, Japan). The plasmid DNA containing the 28S rDNA D8/D10 region of various *Ostreopsis* species was digested with *Eco*RI and *Spa*I before use as a template of qPCR assay. Concentration of the plasmids was measured with DU/730 spectrophotometer (Beckman Coulter, Tokyo, Japan) following the manufacturer’s instruction. An exogenous DNA control, pGEM-3Z (Promega, Tokyo, Japan), was digested with *Nde*I and *Sal*I, quantified as above, and used to determine DNA recovery and/or DNA amplification efficiency.

### Preparation of Genomic DNA for qPCR

**Figure 1:**
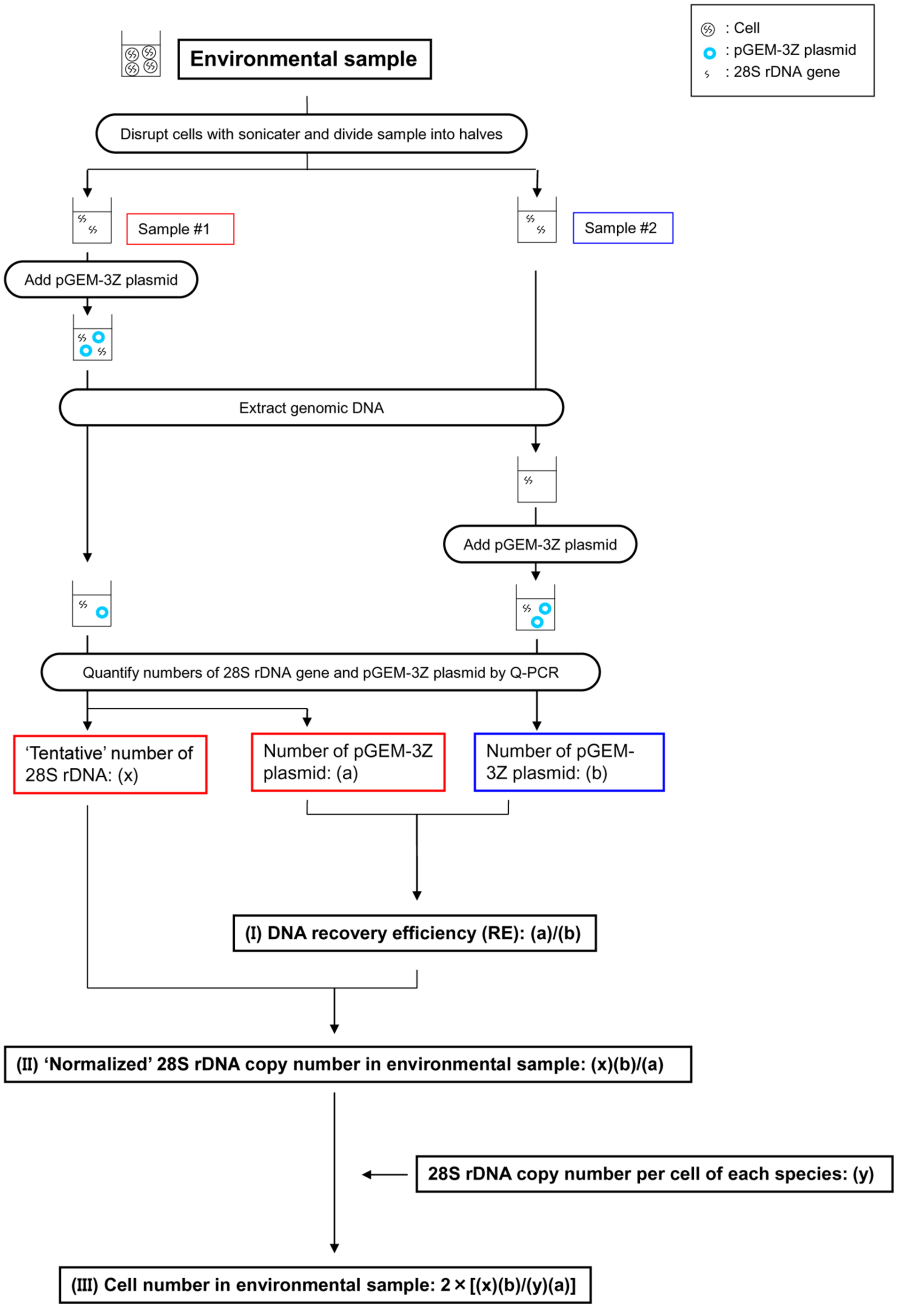
Scheme of the cell quantification method by qPCR used in this study. Normalization with DNA recovery efficiency (RE) by addition of exogenous plasmid DNA was considered in the qPCR method.

Before DNA extraction, cultured cells and environmental samples were sonicated (Handy Sonic UR-20P, Tomy Seiko Co., Tokyo, Japan) in AP1 buffer (Qiagen, Tokyo, Japan) with 10 cycles of 30 sec. sonication and 30 sec. on ice. Sonicated samples were divided into two subsamples, Sample #1 and Sample #2 as shown in Fig. 1. Sample #1 was spiked with pGEM-3Z plasmid before DNA extraction (Fig. 1). Then, DNA was extracted from both samples using DNeasy Plant Mini Kit (Qiagen, Tokyo, Japan) according to the manufacturer’s instructions. Sample #2 was then spiked with pGEM-3Z after DNA extraction (Fig. 1). The DNA extracts were stored at −20°C until the time of qPCR analysis.

### Evaluation of Primer and Probe Specificity

To confirm the specificity of the primer and probe sets listed in Table 2, genomic DNA was extracted from various strains; seven strains of *O.* cf. *ovata*, five strains of *Ostreopsis* sp. 1, two strains of *Ostreopsis* sp. 5, one strain of *Ostreopsis* sp. 6, three strains of *Gambierdiscus* spp. and three strains of *Coolia* sp. (Table 3). pGEM-3Z was also used for the validation of tested primer and probe set specificity. Two µl of the genomic DNA solutions extracted from cells collected from 10 ml of culture sample at the stationary phase and 100,000 copies of pGEM-3Z were used as templates for PCR and qPCR analysis. All samples were analyzed by PCR using 28S rDNA eukaryotic-universal F08 and RB primers [46] and by qPCR with the various *Ostreopsis* species-specific primer and probe sets and pGEM primer and probe set. Amplicons produced with F08 and RB primers were determined by electrophoresis using 1.5% agarose gel (Nippon gene, Tokyo, Japan).

**Table 3:** Cross reactivity of all primer and probe sets.

		PCR*	Q-PCR**				
		Primer	Primer and probe sets				
Template	Strain	F08-RB***	*O*. cf. *ovata*	*Ostreopsis* sp. 1	*Ostreopsis* sp. 5	*Ostreopsis* sp. 6	pGEM
*Ostreopsis* cf. *ovata*	s0579	**+**	**+**	**-**	**-**	**-**	**-**
*Ostreopsis* cf. *ovata*	s0619	**+**	**+**	**-**	**-**	**-**	**-**
*Ostreopsis* cf. *ovata*	s0662	**+**	**+**	**-**	**-**	**-**	**-**
*Ostreopsis* cf. *ovata*	s0667	**+**	**+**	**-**	**-**	**-**	**-**
*Ostreopsis* cf. *ovata*	s0711	**+**	**+**	**-**	**-**	**-**	**-**
*Ostreopsis* cf. *ovata*	s0713	**+**	**+**	**-**	**-**	**-**	**-**
*Ostreopsis* cf. *ovata*	s0731	**+**	**+**	**-**	**-**	**-**	**-**
*Ostreopsis* sp. 1	s0772	**+**	**-**	**+**	**-**	**-**	**-**
*Ostreopsis* sp. 1	s0618	**+**	**-**	**+**	**-**	**-**	**-**
*Ostreopsis* sp. 1	s0659	**+**	**-**	**+**	**-**	**-**	**-**
*Ostreopsis* sp. 1	s0716	**+**	**-**	**+**	**-**	**-**	**-**
*Ostreopsis* sp. 1	s0737	**+**	**-**	**+**	**-**	**-**	**-**
*Ostreopsis* sp. 5	o07421-2	**+**	**-**	**-**	**+**	**-**	**-**
*Ostreopsis* sp. 5	s0577	**+**	**-**	**-**	**+**	**-**	**-**
*Ostreopsis* sp. 6	s0587	**+**	**-**	**-**	**-**	**+**	**-**
*Gambierdiscus* sp. type 1	KW070922_1	**+**	**-**	**-**	**-**	**-**	**-**
*Gambierdiscus* sp. type 2	T070411_1	**+**	**-**	**-**	**-**	**-**	**-**
*Gambierdiscus australes*	I080606_1	**+**	**-**	**-**	**-**	**-**	**-**
*Coolia* sp.	S32C	**+**	**-**	**-**	**-**	**-**	**-**
*Coolia* sp.	S33C	**+**	**-**	**-**	**-**	**-**	**-**
*Coolia* sp.	S34C	**+**	**-**	**-**	**-**	**-**	**-**
pGEM-3Z plasmid		**-**	**-**	**-**	**-**	**-**	**+**
NTC (distilled water)		**-**	**-**	**-**	**-**	**-**	**-**

* PCR amplification was determined by electrophoresis using 1.5% agarose gel.

** PCR amplification was determined by Q-PCR.

*** F08-RB indicate eukaryotic universal primers [46].

### Evaluation of Sensitivity of Primer and Probe Sets

Standard curves for each species-specific primer and probe set were constructed with a 10-fold dilution series (from 10^7^ to 10^1^ copies) of pMD20 plasmid containing the 28S rDNA D8/D10 regions of each species of *Ostreopsis*, respectively. Standard curves were constructed by using the average Ct values measured in three independent experiments. Error bars with standard curves represent the standard deviation of triplicate PCR reactions. Amplification efficiency (AE) was calculated according to the equation (AE = 10^(−1/slope)^) described by Coyne et al. [12].

### Comparison between AEs of Circular Plasmid and Linear Plasmid Spiked with and without an Environmental Sample

Amplification efficiencies (AEs) of circular pGEM-3Z and linearized pGEM-3Z plasmid alone or spiked with an environmental sample using the pGEM primer and probe set were compared to examine the effect of DNA structural conformation and the environmental sample matrix on qPCR results. Ten-fold dilution series of circular and linearized pGEM-3Z plasmid were used for construction of standard curves (from 10^6^ to 10^1^ copies). Genomic DNA extracted from an environmental sample collected from Muroto, Kochi, Japan (Muroto100828) that contained various diatom cells as well as *Ostreopsis* spp. was prepared following the method described above. For the environmental sample matrix test, two µl of the DNA solution extracted from the environmental sample (Muroto100828) was added to qPCR reaction. Standard curves for each were determined as described above. Error bars represent standard deviation of triplicate PCR reactions.

Standard curves of various *Ostreopsis* species- specific primer and probe sets were constructed with a 10-fold dilution series of linearized pMD20 plasmid containing the 28S rDNA D8/D10 regions of the target species (from 10^6^ to 10^1^ copies) alone or spiked with DNA from an environmental sample (Maizuru100904) which was collected from Maizuru, Kyoto, Japan and did not contain any cells of *Ostreopsis* spp. The amplification efficiency (AEs) was determined according to the method described above. Error bars represent standard deviation of triplicate PCR reactions. Statistical analyses were performed using Student t-test between amplification efficiencies with species-specific primer and probe set and that with pGEM primer and probe set. Significance was accepted at *p*<0.05.

### Comparison between AE of Genomic DNA of Cultured *Ostreopsis* Spiked with DNA from an Environmental Sample and AE of Exogenous Control Plasmid

1.37×10^6^ cells of *O.* cf. *ovata* (s0662), 1.76×10^6^ cells of *Ostreopsis* sp. 1 (s0716), 1.86×10^5^ cells of *Ostreopsis* sp. 5 (o07421-2) and 2.50×10^6^ cells of *Ostreopsis* sp. 6 (s0587) were harvested from cultures, sonicated and spiked with 4.0×10^9^ copies of linearized pGEM-3Z plasmid. Then, the genomic DNA was extracted according to the method described above. A ten-fold dilution series of the genomic DNA were prepared and then spiked with 2 µl of DNA solution extracted from the environmental sample (Maizuru100904). Using the dilution series as templates, qPCR was conducted with *Ostreopsis* species-specific primer and probe set and pGEM primer and probe set. Standard curves were constructed using the average Ct values of triplicate reactions. Error bars represent standard deviation of triplicate PCR reactions. Statistical analysis was performed using Student t-test between AE of genomic DNA of each *Ostreopsis* spp. and AE of pGEM-3Z plasmid. Significance was accepted at *p*<0.05.

### Validation of Normalization with DNA Recovery Efficiency

A sample which contains 1.5×10^7^ copies of pMD20 plasmid containing the 28S rDNA D8/D10 region of *O*. cf. *ovata* and a sample which contains 2.0×10^7^ copies of plasmid containing of *Ostreopsis* sp. 5 were prepared, respectively. Two samples with the 28S rDNA D8/D10 region in pMD20 containing either 1.5×10^6^ copies of *O*. cf. *ovata* or 2.0×10^6^ copies *Ostreopsis* sp. 5 were spiked into sample Maizuru100904 and sonicated. The two samples were divided into two subsamples (Sample #1 and Sample #2, as shown in Fig. 1). Sample #1 was spiked with pGEM-3Z plasmid before DNA extraction. DNA was extracted from Sample #1 and Sample #2 by the method described above, respectively. After DNA extraction, DNA from Sample #2 was spiked with pGEM-3Z plasmid. The number of plasmids containing the 28S rDNA D8/D10 region of the target species in Sample #1 was estimated using qPCR and then normalized with ‘DNA recovery efficiency’ (RE). In order to determine RE, Ct values of Sample #1 and Sample #2 were determined by qPCR using pGEM primer and probe set. Copy number of pGEM-3Z in Sample #1 and Sample #2 (a and b, respectfully, Fig. 1) were calculated by interpolation of Ct experimentally determined on the standard curve of pGEM-3Z. Extraction efficiency was calculated as follows:
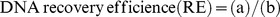
(1)


In order to determine the number of plasmid containing the 28S rDNA of the target species, Ct value of Sample #1 was determined by qPCR using species-specific primer and probe sets according to the method described above. The ‘tentative’ number of 28S rDNA in Sample #1 ((x) in Fig. 1) was calculated by interpolation of Ct experimentally determined on the standard curve. ‘Normalized’ number of 28S rDNA copy in the sample was calculated considering RE as follows:
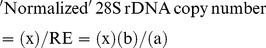
(2)


The ‘tentative’ number of 28S rDNA (x), the ‘normalized’ 28S rDNA copy number [(x)(b)/(a)] and the ‘actual’ copy number of plasmid that was added to samples were compared. Statistical analysis was performed using Student t-test between quantification results with or without normalization considering DNA recovery efficiency. Significance was accepted at *p*<0.05.

### Quantification of 28S rDNA Copy Numbers per Cell of Various *Ostreopsis* Species

To quantify the copy number of the 28S rDNA gene per cell for each *Ostreopsis* species, ten cells of each species were isolated from cultures of each species and environmental samples by micropipetting under microscopy. Each cell was washed with distilled f/2 medium three times and isolated in a 1.5 ml sampling tube. Because *O.* cf. *ovata* and *Ostreopsis* sp. 1 are dominant species in Japanese coastal waters [44], five Japanese cultures and three environmental samples for *O*. cf. *ovata* and three cultures and seven environmental samples for *Ostreopsis* sp. 1 were used for isolation of ten cells (Table 4). Genomic DNA was isolated from each cell according to the method described above. The 28S rDNA copy number of each cell determined by qPCR considering normalization with RE as described above. The number of rDNA gene copy per cell of each culture and environmental sample were determined as the average number of cellular rDNA copies from ten single cells isolated from culture samples or environmental samples. Number of rDNA copy of each *Ostreopsis* species was determined as the average number of rDNA copies from single cells isolated from both cultures and the environmental samples.

**Table 4:** 28S rDNA copy numbers per cell of *Ostreopsis* species of cultures and environmental samples.

Species	Sample	Sample name	rDNA copies cell^-1^ (n = 10)	Averaged rDNA copies of *Ostreopsis* species cell^-1^	CV_copy_%
*Ostreopsis* cf. *ovata*	Culture	s0593	24,000±3,000	24,000±5,000	20.8
*Ostreopsis* cf. *ovata*	Culture	s0662	24,000±7,000		
*Ostreopsis* cf. *ovata*	Culture	s0743	20,000±9,000		
*Ostreopsis* cf. *ovata*	Culture	s0579	28,000±10,000		
*Ostreopsis* cf. *ovata*	Culture	s0619	17,000±30,00		
*Ostreopsis* cf. *ovata*	Environmental sample	Ishigaki110730	43,000±19,000	36,000±8,000	22.2
*Ostreopsis cf. ovata*	Environmental sample	Chiba110912	38,000±15,000		
*Ostreopsis* cf. *ovata*	Environmental sample	Uruma110825	27,000±14,000		
*Ostreopsis* sp. 1	Culture	s0618	67,000±9,000	58,000±12,000	20.7
*Ostreopsis* sp. 1	Culture	s0716	44,000±11,000		
*Ostreopsis* sp. 1	Culture	s0737	62,000±13,000		
*Ostreopsis* sp. 1	Environmental sample	Tei110419	71,000±16,000	88,000±22,000	25.0
*Ostreopsis* sp. 1	Environmental sample	Susaki11110420	90,000±44,000		
*Ostreopsis* sp. 1	Environmental sample	WTK110521	120,000±16,000		
*Ostreopsis* sp. 1	Environmental sample	TI110518	73,000±18,000		
*Ostreopsis* sp. 1	Environmental sample	Miyazaki110813	75,000±29,000		
*Ostreopsis* sp. 1	Environmental sample	Hokkaido110828	120,000±30,000		
*Ostreopsis* sp. 1	Environmental sample	Niigata111014	67,000±11,000		
*Ostreopsis* sp. 5	Culture	o07421-2	500,000±90,000	500,000±90,000	18.0
*Ostreopsis* sp. 5	Environmental sample	Kochi100924	390,000±100,000	390,000±100,000	25.6
*Ostreopsis* sp. 6	Culture	ISC06	200,000±40,000	270,000±160,000	59.3
*Ostreopsis* sp. 6	Culture	ISC07	160,000±60,000		
*Ostreopsis* sp. 6	Culture	s0587	460,000±100,000		
*Ostreopsis* sp. 6	Environmental sample	Kochi100924	340,000±110,000	320,000±180,000	56.3
*Ostreopsis* sp. 6	Environmental sample	Ishigaki110730	310,000±160,000		

### Cell Quantification of Each *Ostreopsis* Species in Mixed Samples Containing Cultured Cells of Various Species

To confirm the accuracy and specificity of qPCR considering normalization with RE, mixed samples of cultured cells of various *Ostreopsis* species were prepared as follows:

Sample A contained 1,000±100 cells of cultured *O*. cf. *ovata* (s0662), 1,200±80 cells of *Ostreopsis* sp. 1 (s0716), 2,100±400 cells of *Ostreopsis* sp. 5 (o07421-2) and 2,200±200 cells of *Ostreopsis* sp. 6 (s0587). Sample B contained 170,000±20,000 cells of cultured *O*. cf. *ovata* (s0662), 150±10 cells of *Ostreopsis* sp. 1 (s0716), 90±30 cells of *Ostreopsis* sp. 5 (o07421-2) and 100±30 cells of *Ostreopsis* sp. 6 (s0577). Sample C contained 150±20 cells of cultured *O*. cf. *ovata* (s0662), 100,000±30,000 cells of *Ostreopsis* sp. 1 (s0716), 90±30 cells of *Ostreopsis* sp. 5 (o07421-2) and 100±30 cells of *Ostreopsis* sp. 6 (s0587). Cells in these samples were sonicated and divided into two sub-samples, Sample #1 and Sample #2 as described in Fig. 1. Genomic DNA of these samples was extracted following the method described above. These DNA samples were spiked with the previously extracted environmental sample (Maizuru100904) which did not contain any cells of *Ostreopsis* spp. Number of 28S rDNA copies in Sample #1 was quantified by qPCR considering normalization with RE as described above. Number of cells in each subsample was calculated as follows:
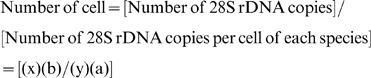
(3)(y) represents the number of 28S rDNA copies per cell of each *Ostreopsis* species determined by the method described above.

### Cell Enumeration of *Ostreopsis* Species in Environmental Samples

A total of six environmental samples (Nos. 1–6, Table 5) containing cells of *Ostreopsis* spp. were collected from Japanese coastal waters for cell enumeration of *Ostreopsis* species by qPCR considering normalization with RE. Epiphytic microalgal samples were prepared following the protocols for environmental samples as described above. The number of cells of *Ostreopsis* spp. in the samples were counted under an inverted microscope (Olympus, Tokyo, Japan). Fresh weight of macroalgal samples was measured ( = z g). The detached microalgal samples from the macroalgal samples were sonicated and divided into two sub-samples (Sample #1 and Sample #2) as shown in Fig. 1. The normalized number of 28S rDNA copies of each *Ostreopsis* species in the samples were determined by qPCR considering normalization with RE as described above. Number of cells in each sample was calculated as follows:
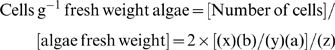
(4)


**Table 5:** List of environmental samples used in experiments to enumerate *Ostreopsis* spp. using qPCR.

Sample number	Locality	Latitude	Longitude	Sampling date	Water temperature (°C )
No.1	Kashiwajima, Otsuki Town, Kochi Pref.	32.8	132.6	9/24/2011	27.7
No.2	Kushimoto Kamiura, Kushimoto Town, Wakayama Pref.	33.5	135.8	10/4/2011	25.3
No.3	Tei, Konan City, Kochi Pref.	31.9	131.5	8/29/2011	29.7
No.4	Ishigakijima Island, Touzato, Ishigaki City, Okinawa Pref.	24.4	124.3	7/30/2011	32.4
No.5	Ishigakijima Island, Ishigaki City, Okinawa Pref.	24.4	124.3	9/10/2011	31.5
No.6	Uken beach, Uruma City, Okinawa Pref.	26.3	127.9	8/28/2011	32.0

## Results

### Assay Specificity

For development of *Ostreopsis* species-specific quantification method applicable to environmental samples, *Ostreopsis* species-specific primer and probe sets for qPCR were designed to target the D8/D10 region of 28S rDNA of *Ostreopsis* (Table 2). The specificity of primers and probes were evaluated against DNA extracted from various cultivated strains of *Ostreopsis*, *Gambierdiscus* and *Coolia* (Table 3). The qPCR amplification demonstrated no cross reactivity when DNAs from non-target species were used as templates (Table 3). In contrast, DNA was amplified from the non-target strains as demonstrated by positive PCR amplification of D8/D10 region of 28S rDNA using eukaryotic-universal primers F08-RB. In addition, species-specific primer and probe sets only amplified their target strains indicating the assays could distinguish different *Ostreopsis* clades. All primer and probe sets showed no amplification for NTC reactions when distilled water was used as a template (Table 3). The reproducibility listed in Table S1 was evaluated by calculating the CV_Ct_ (Coefficient of variation of cycle threshold) using a dilution series of culture cells. The CVct mean values for the primer and probe sets were 0.5%, 0.4%, 0.7% and 0.6% for *O*. cf. *ovata*, *Ostreopsis* sp. 1, *Ostreopsis* sp. 5 and *Ostreopsis* sp. 6, respectively. No amplification was detected with the pGEM-3Z plasmid primer and probe set using genomic DNA from all strains of *Ostreopsis*, *Coolia* and *Gambierdiscus.* In addition, only the pGEM primer and probe set yielded positive amplification with the pGEM-3Z plasmid; none of the genus or strain specific *Ostreopsis* primer and probe set amplified (Table 3).

### AEs of Circular and Linearized Control Plasmid Spiked with or without DNA Extracted from an Environmental Sample

**Figure 2:**
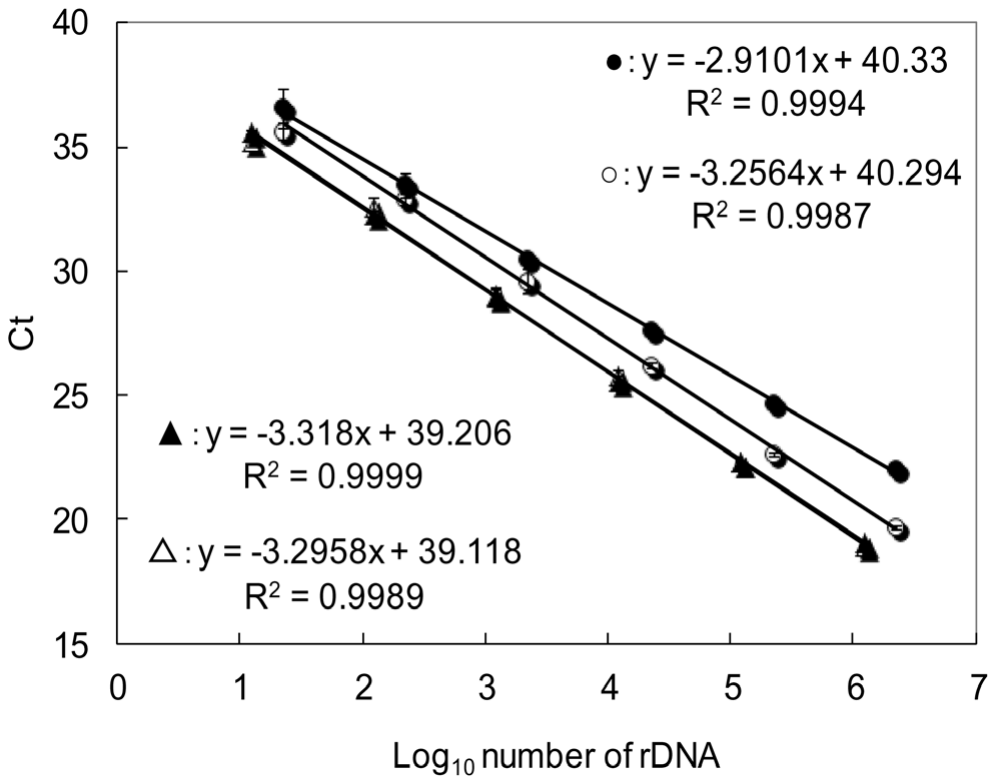
Standard curves of circular and linear plasmid with or without the addition of an environmental sample. Standard curves of pGEM-3z assay generated by circular and linearized pGEM-3Z plasmid with or without the addition of an environmental sample were generated. Error bars represent standard deviation of triplicate PCR reactions. Black circle: circular pGEM-3Z with an environmental sample. Open circle: circular pGEM-3Z. Black triangle: linear pGEM-3Z with the environmental sample. Open triangle: linearized pGEM-3Z.

Standard curves generated from circular and linearized pGEM-3Z with or without the addition of DNA extracted from an environmental sample containing epiphytic microorganisms are shown in Fig. 2. The detection limit for pGEM-3Z primer and probe set was about 10 copies with a linear range of detection over 6 orders of magnitude, *R^2^* = 0.99 (Fig. 2). A significant difference (P<0.05) was observed between AEs of circular pGEM-3Z plasmid spiked with (2.21) and without (2.03) DNA extracted from the environmental sample (Muroto100828, Fig. 2). In contrast, no difference was observed between AEs of linearized plasmid spiked with and without DNA extracted from the environmental sample, 2.00 and 2.01 (*p*>0.05), respectively.

### Standard Curves, Dynamic Range and AE of the Assays

**Figure 3:**
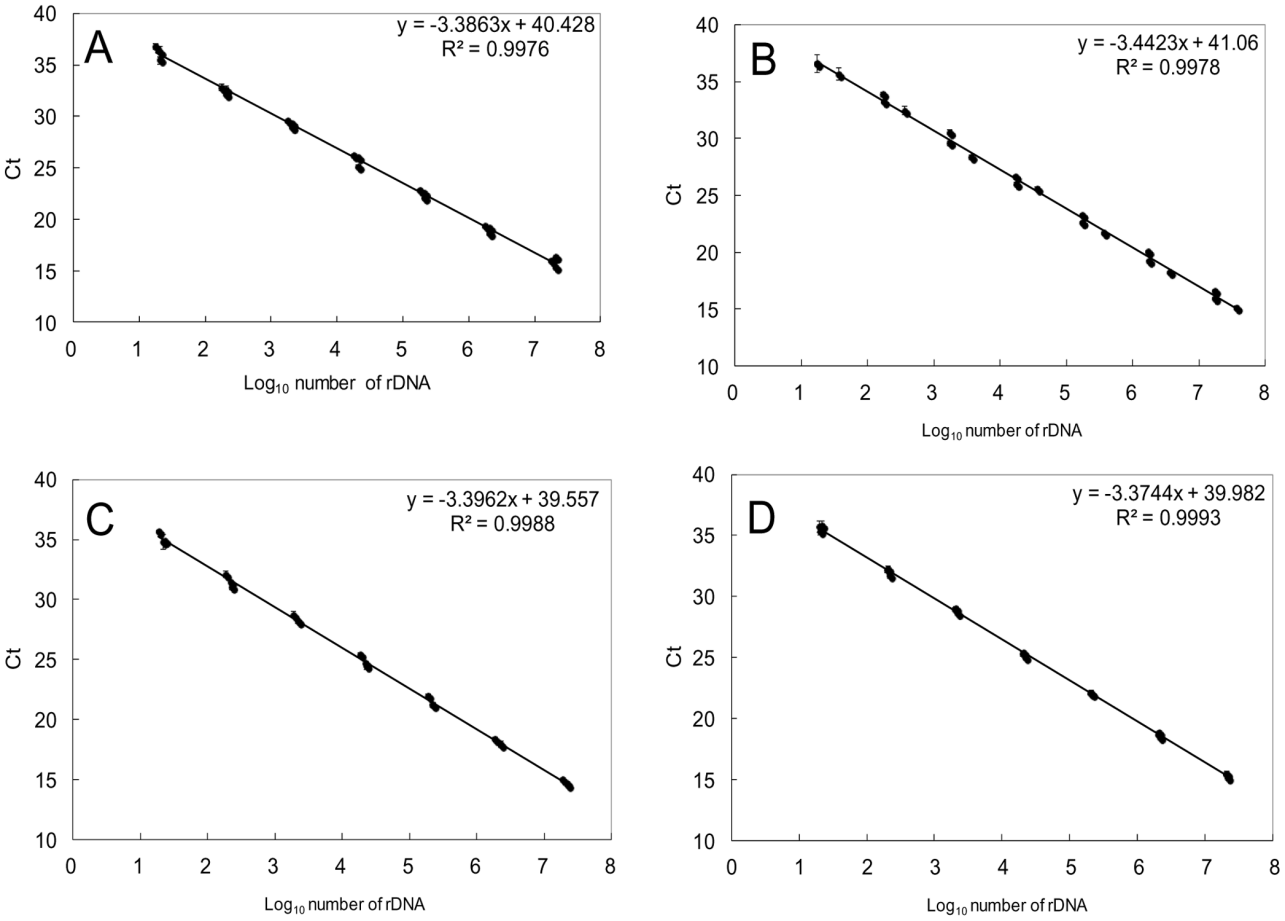
Dynamic range and sensitivity of the qPCR assay. Standard curves were generated by plasmids containing the 28S rDNA D8/D10 sequences of various species of *Ostreopsis*. Each standard curve was generated by the plasmid containing the target sequence of 28S rDNA D8/D10 using *O*. cf. *ovata* primer and probe set (A), *Ostreopsis* sp. 1 primer and probe set (B), *Ostreopsis* sp. 5 primer and probe set (C) *Ostreopsis* sp. 6 primer and probe set (D). Error bars represent standard deviation of triplicate PCR reactions.

The detection limits for all primer and probe sets used in this study were approximately 10 copies with a linear range of detection over 7 orders of magnitude, *R^2^*>0.99, (Fig. 3). Amplification efficiencies (AEs) of the primer and probe sets were 1.95, 1.94, 1.95 and 1.97 for *O.* cf. *ovata*, *Ostreopsis* sp. 1, *Ostreopsis* sp. 5 and *Ostreopsis* sp. 6, respectively.

**Figure 4:**
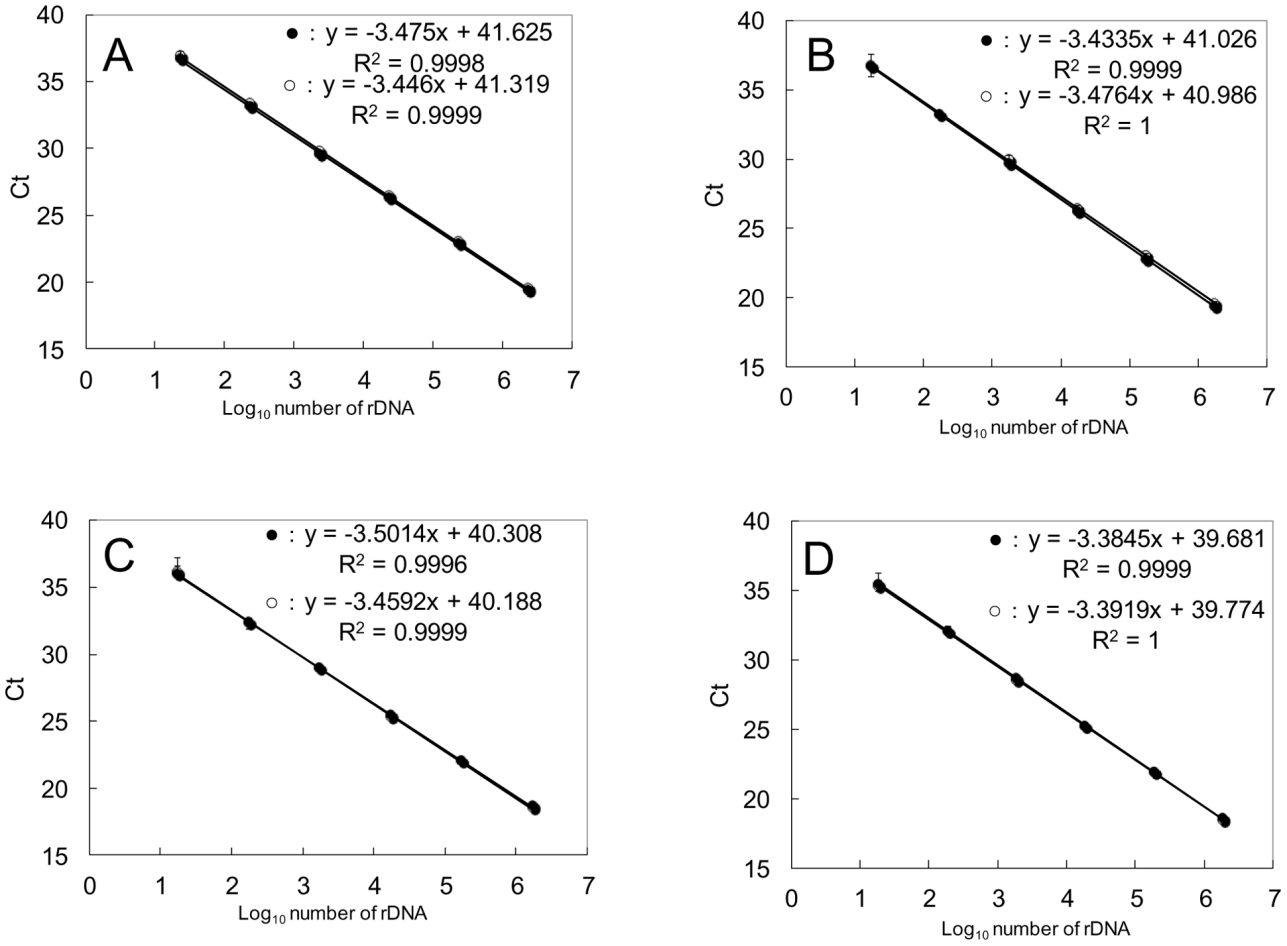
Standard curves of linear plasmid spiked with or without an environmental sample. Standard curves of linear target plasmid containing the 28S rDNA spiked with DNA from the environmental sample (Maizuru100904) using various *Ostreopsis* species-specific primer and probe sets (A: *O*. cf. *ovata*; B: *Ostreopsis* sp. 1; C: *Ostreopsis* sp. 5; D: *Ostreopsis* sp. 6) were constructed with a 10-fold dilution series of the plasmid. Error bars represent standard deviation of triplicate PCR reactions. Black circle: plasmid containing the 28S rDNA spiked with DNA from the environmental sample. Open circle: plasmid containing the 28S rDNA without DNA from the environmental sample.

We determined AEs of linearized plasmids containing the 28S rDNA D8/D10 region of the four species of *Ostreopsis* spiked with and without DNA extracted from the environmental sample (Maizuru100904), which not to contain *Ostreopsis*. AEs using plasmid DNA only for *O*. cf. *ovata*, *Ostreopsis* sp. 1, *Ostreopsis* sp. 5 and *Ostreopsis* sp. 6 (Fig. 4) were 1.94, 1.96, 1.93 and 1.98, respectively. AEs of plasmid DNA with the addition of an environmental sample for *O*. cf. *ovata*, *Ostreopsis* sp. 1, *Ostreopsis* sp. 5 and *Ostreopsis* sp. 6 were 1.95, 1.94, 1.95 and 1.97, respectively (Fig. 4). In all assays, no significant difference was observed between amplification efficiencies of the plasmids spiked with and without DNA from the environmental sample (*p*>0.05,Fig. 4). The AEs were not significantly different from that (AE = 1.98) of linearized control pGEM-3Z plasmid spiked with DNA from the environmental sample (*p*>0.05; data not shown).

### AEs of Genomic DNA of *Ostreopsis* Spiked with DNA from an Environmental Sample and of Exogenous Control Plasmid

**Figure 5:**
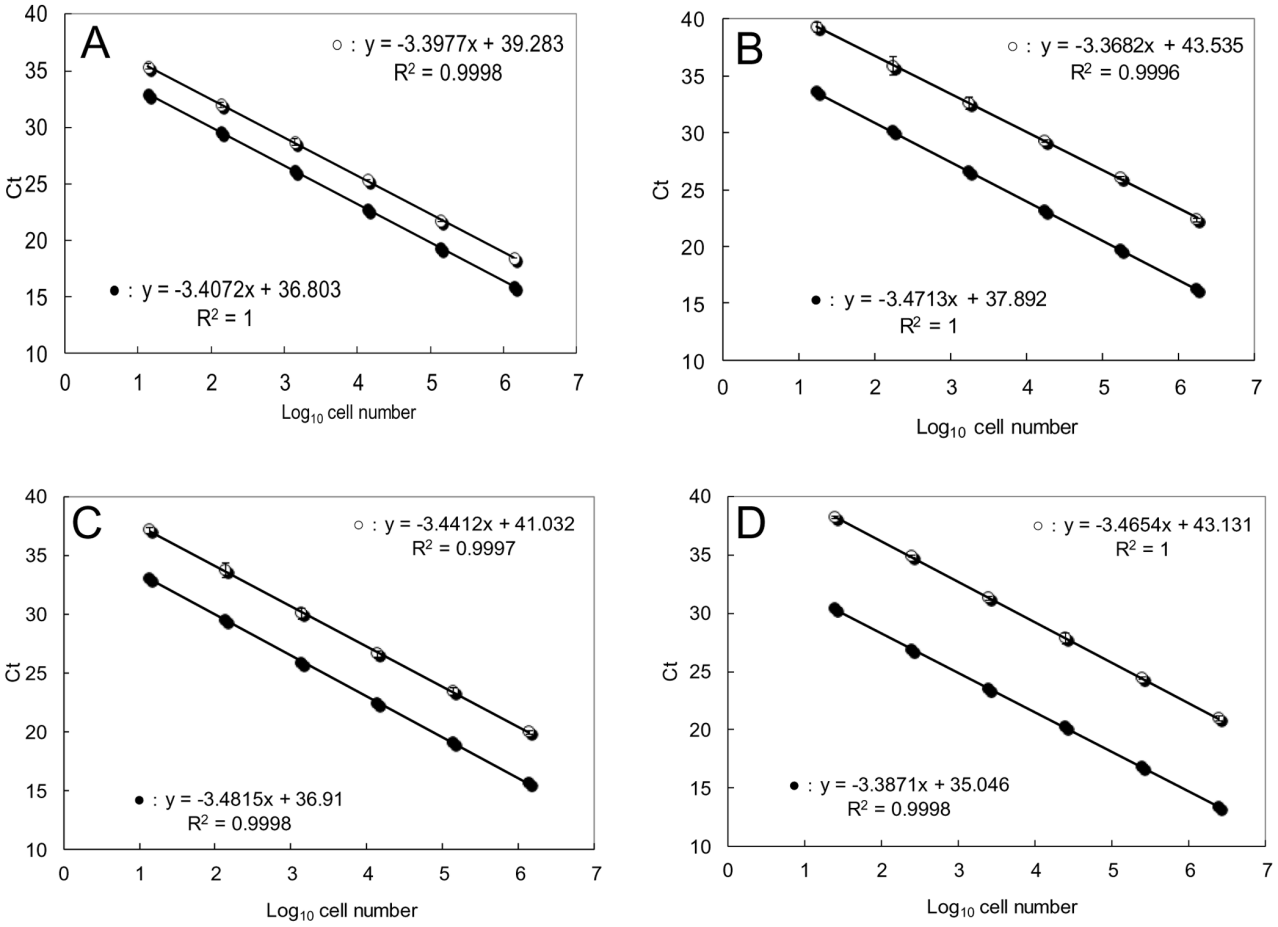
Standard curves of *Ostreopsis* genomic DNA and control plasmid. Cells of the four *Ostreopsis* species were harvested from cultures, sonicated and spiked with 4.0×10^9^ copies of linearized pGEM-3Z plasmid, respectively. Then, the genomic DNA were extracted. Ten-fold dilution series of the genomic DNA were prepared and then spiked with 2 µl of DNA solution extracted from the environmental sample (Maizuru100904). Using the dilution series as templates, qPCR was conducted with *Ostreopsis* species-specific primer and probe set (Black circle) and pGEM primer and probe set (Open circle). Error bars represent standard deviation of triplicate PCR reactions. Standard curves of *O.* cf. *ovata* (A), *Ostreopsis* sp. 1 (B), *Ostreopsis* sp. 5 (C) and *Ostreopsis* sp.6 (D).

Standard curves obtained using genomic DNA of *Ostreopsis* species and pGEM spiked before DNA extraction are shown in Fig. 5. AEs of genomic DNA of strains of *O*. cf. *ovata*, *Ostreopsis* sp. 1, *Ostreopsis* sp. 5 and *Ostreopsis* sp. 6 (Fig. 5) were 1.96, 1.94, 1.93 and 1.97, respectively. AEs of pGEM-3Z in each assay were 1.96, 1.98, 1.95 and 1.94, respectively. The former AEs were not significantly different from the later AEs and were not different from that of plasmid only as shown in Fig. 2.

### Validation of Normalization with ‘DNA Recovery Efficiency’ (RE)

**Figure 6:**
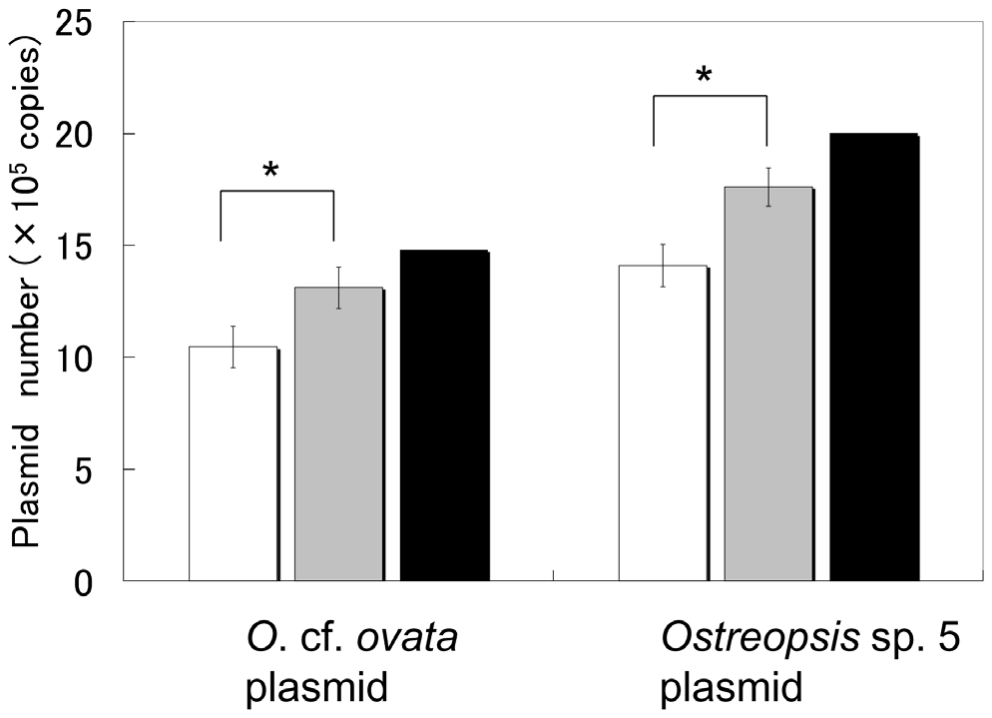
Validation of normalization with DNA recovery efficiency. Comparison of the ‘tentative’ number of 28S rDNA copies determined by qPCR (White column), ‘normalized’ number of 28S rDNA copies determined by qPCR normalized with DNA recovery efficiency (Grey column) and ‘actual’ number of copies (1.50×10^6^ and 2.00×10^6^ molecules) of added plasmid containing the 28S rDNA D8/D10 sequences of *O*. cf. *ovata* and *Ostreopsis* sp. 5 (Black column). Values are mean ± SD. Asterisk show significant difference between qPCR results without normalization and that with normalization by DNA recovery efficiency (*p*<0.05).

The ‘tentative’ number of the 28S rDNA ( = (x), Sample #1 in Fig. 1) of *O*. cf. *ovata* and *Ostreopsis* sp. 5 as determined by qPCR was 1.05×10^6^ and 1.41×10^6^ copies, respectively, in the *Ostreopsis*-free environmental sample (Maizuru100904) where 1.50×10^6^ molecules of the plasmid containing *O*. cf. *ovata* and 2.00×10^6^ molecules of the plasmid containing *Ostreopsis* sp. 5 were added (Fig. 6). DNA recovery efficiencies (REs) of the samples that were calculated using Equation (I) (Fig. 1) were 0.80 and 0.79, respectively. The ‘normalized’ number of 28S rDNA copies in these samples were 1.31×10^6^ and 1.99×10^6^ calculated by Equation (II), respectively (Fig. 6). The normalized number of copies were significantly higher (*p*<0.01) and were close to the number of 28S rDNA genes of *O*. cf. *ovata* and *Ostreopsis* sp. 5 added to Sample #1 and Sample #2 (Fig. 6).

### Quantification of the Number of 28S rDNA Copies of *Ostreopsis* Species

For quantification of the number of copies of 28S rDNA per cell of each species, five strains of *O.* cf. *ovata*, three strains of *Ostreopsis* sp. 1, one strain of *Ostreopsis* sp. 5 and three strains of *Ostreopsis* sp. 6 were analyzed using species-specific probe and primer sets. The range of number of 28S rDNA copies per cell of *O*. cf. *ovata*, *Ostreopsis* sp. 1, *Ostreopsis* sp. 5 and *Ostreopsis* sp. 6 in a cultured cell were 17,000–28,000 copies, 44,000–67,000 copies, 500,000 copies and 160,000–460,000 copies, respectively. The average number of 28S rDNA copies of cultured *O*. cf. *ovata*, *Ostreopsis* sp. 1, *Ostreopsis* sp. 5 and *Ostreopsis* sp. 6 were 24,000±5,000, 58,000±12,000, 500,000±90,000 and 270,000±160,000 copies per cell, respectively (Table 4). The reproducibility was evaluated by calculating the CV_copy_ (coefficient of variation of 28S rDNA copies/cell). The CV_copy_ mean values of each *Ostreopsis* spp. in culture were 20.8% for *O*. cf. *ovata*, 20.7% for *Ostreopsis* sp. 1, 18.0% for *Ostreopsis* sp. 5 and 59.3% for *Ostreopsis* sp. 6.

The average copy numbers of 28S rDNA of the four species in 13 environmental samples analyzed by using species-specific probe and primer sets were 36,000±8,000, 88,000±22,000, 390,000±100,000 and 320,000±180,000 (Table 4). The CV_copy_ mean values of each *Ostreopsis* spp. isolated from environmental samples were 22.2% for *O*. cf. *ovata*, 25.0% for *Ostreopsis* sp. 1, 25.6% for *Ostreopsis* sp. 5 and 56.3% for *Ostreopsis* sp. 6. The average number of 28S rDNA copies per cell of *O*. cf. *ovata*, *Ostreopsis* sp. 1 and *Ostreopsis* sp. 6 in environmental samples tended to be more than that in cultured samples. No significant difference was detected among number of rDNA copies of cells from cultures and those from environmental samples (*p*>0.05).

### Validation of Number of 28S rDNA Copies per Cell among Growth Phases

The 28S rDNA copy number of *Ostreopsis* sp. 1 at various growth phases was analyzed by qPCR considering normalization with RE described above. Single cells in cultured *Ostreopsis* sp. 1 (s0716) were isolated at early exponential growth phase (6^th^ day after inoculation), exponential growth phase (19^th^ day after inoculation) and stationary phase (26^th^ day after inoculation). Average number of 28S rDNA copies in each growth phase showed about 71,000±9,000 copies in early exponential growth phase, 59,000±7,000 copies in exponential growth phase, and 51,000±13,000 copies in stationary phase (Fig. S1). No significant difference was detected among average 28S rDNA copy numbers among the three growth phases (*p*>0.05).

### Cell Quantification of Each *Ostreopsis* Species in Mixed Samples of Cultured Cells of Various Species

**Figure 7:**
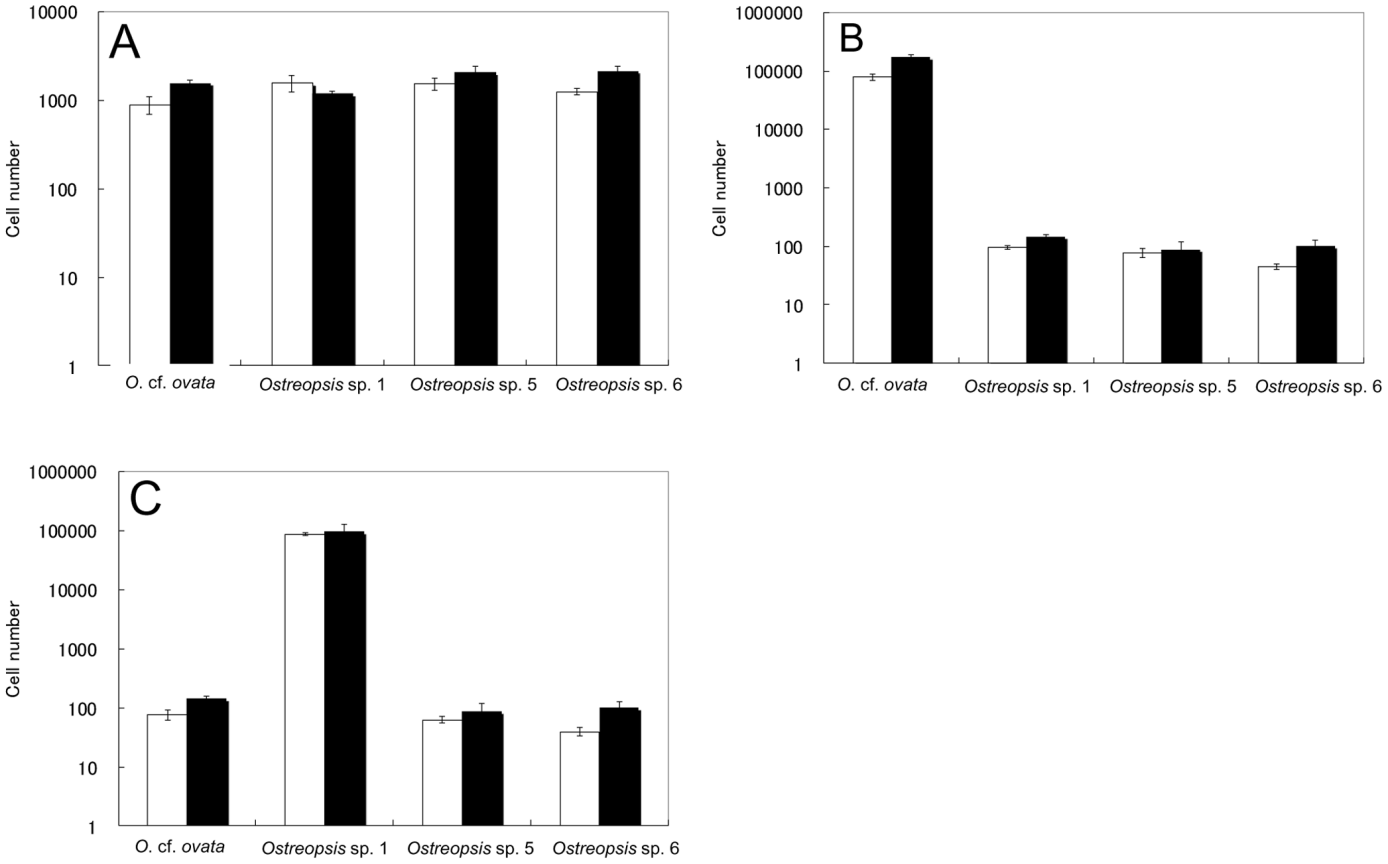
Cell quantification of each species in mixed samples of cultured cells of various species. Cell quantification of each *Ostreopsis* species in three mixed culture samples (A–C) spiked with the environmental sample (Maizuru100904) by qPCR considering normalization with RE. A: qPCR quantification result of four species in mixed culture sample (Sample A) containing 900±200 cells of cultured *O.* cf. *ovata,* 1,500±350 cells of *Ostreopsis* sp. 1, 1,500±230 cells of *Ostreopsis* sp. 5 and 1,260±110 cells of *Ostreopsis* sp. 6. B: qPCR result in Sample B containing 90,000±5,000 cells of cultured *O.* cf. *ovata,* 100±10 cells of *Ostreopsis* sp. 1, 80±10 cells of *Ostreopsis* sp. 5 and 50±5 cells of *Ostreopsis* sp. 6. C: qPCR result in Sample C containing *O.* cf. *ovata,* 80,000±4,000 cells of *Ostreopsis* sp. 1, 70±10 cells of *Ostreopsis* sp. 5 and 50±10 cells of *Ostreopsis* sp. 6. White columns: cell number determined by qPCR method considering normalization with RE, Black columns: actual number of cells spiked in environmental sample. Numbers on x-axis represented Log_10_ cell number. Values are mean ± SD.

To confirm the accuracy and specificity of the qPCR method, mixed samples which contained various numbers of cultured cells of the four species of *Ostreopsis* were analyzed by qPCR considering normalization with RE. The average rDNA copy numbers per cell of *O*. cf. *ovata*, *Ostreopsis* sp. 1, *Ostreopsis* sp. 5 and *Ostreopsis* sp. 6 in environmental samples shown in Table 4 were used to estimate the number of cells of each species in the mixed samples of cultured cells (Samples A–C). When Sample A was analyzed by the qPCR considering normalization with RE, the cell quantification results were 900±200 cells of cultured *O.* cf. *ovata,* 1,500±350 cells of *Ostreopsis* sp. 1, 1,500±230 cells of *Ostreopsis* sp. 5 and 1,300±100 cells of *Ostreopsis* sp. 6 (Fig. 7A). When Sample B was analyzed, the cell quantification results the qPCR method were 90,000±5,000 cells of cultured *O.* cf. *ovata,* 100±10 cells of *Ostreopsis* sp. 1, 80±10 cells of *Ostreopsis* sp. 5 and 50±5 cells of *Ostreopsis* sp. 6 (Fig. 7B). In Sample C, the cell quantification results using the qPCR method were 80±20 cells of cultured *O.* cf. *ovata,* 80,000±4,000 cells of *Ostreopsis* sp. 1, 70±10 cells of *Ostreopsis* sp. 5 and 50±10 cells of *Ostreopsis* sp. 6 (Fig. 7C). There was no significant difference between the actual numbers added and those estimated by the qPCR method (p>0.05).

### Cell Enumeration of *Ostreopsis* Species in Environmental Samples

Cells of *Ostreopsis* spp. in the six environmental samples (Nos. 1–6, Table 5) were counted by microscopy and cells of the four species of *Ostreopsis* were quantified using the qPCR method considering normalization with RE. In advance of cell quantification, we determined AEs of *O*. cf. *ovata*, *Ostreopsis* sp. 1, *Ostreopsis* sp. 5 and *Ostreopsis* sp. 6 in environmental samples of Nos. 1–4, respectively (Fig. S2). No difference was observed between AEs of the genomic DNA extracted from the samples and those of the linearized pGEM-3Z spiked before DNA extraction (*p*>0.05).

**Figure 8:**
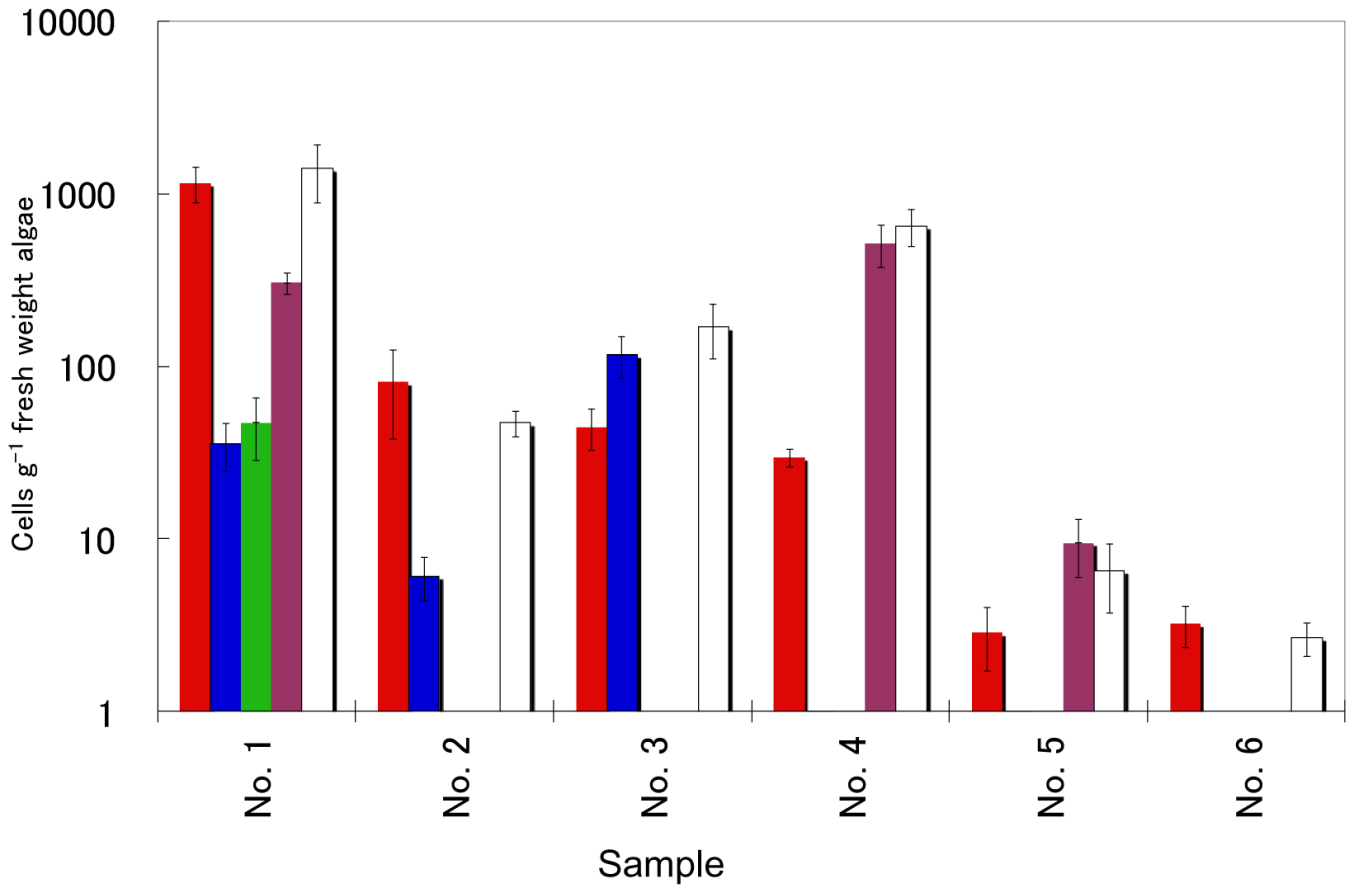
Cell enumeration of *Ostreopsis* species in environmental samples. Number of cells of four species of *Ostreopsis* were determined by qPCR considering normalization with RE in environmental samples collected from various locals of Japanese coastal waters listed in Table 5. Cell number of *O*. cf. *ovata* (red), *Ostreopsis* sp. 1 (blue), *Ostreopsis* sp. 5 (green), *Ostreopsis* sp. 6 (purple) as determined by qPCR with RE. Total cell number of *Ostreopsis* species counted by microscopy (white). Numbers on y-axis represented Log_10_ cells g^−1^ fresh weight algae. Values are mean ± SD.

In most of the samples, multiple species of *Ostreopsis* were detected. Cells of both *O*. cf. *ovata* and *Ostreopsis* sp. 1 which are cryptic species of *O. ovata* were detected in sample Nos. 1, 2 and 3, which were collected from the temperate region, Shikoku, in Japan (Fig. 8). Samples Nos. 4 and 5 that were collected from the subtropical region, Okinawa, in Japan, contained *O.* cf. *ovata* and *Ostreopsis* sp. 6 (Fig. 8).

When the numbers of rDNA copies per cultured cell were used for cell enumeration with qPCR method, significant differences between the total numbers of *Ostreopsis* spp. cells determined by microscopy and the total numbers of each cell number of the four species enumerated with qPCR method, respectively, were detected in samples No. 2 and 5 (data not shown). When the number of rDNA copies of each *Ostreopsis* species per cell isolated from environmental samples was used for cell quantification, no significant difference was detected in any of the samples.

## Discussion

The goal of this investigation was to develop a qPCR method that is applicable for detection and enumeration of each target HAB species containing cryptic species in environmental samples. *Ostreopsis* spp. which form blooms in Japanese coastal areas was chosen as the model target species for this study due to their potential impacts on human health. To achieve this goal, high specificity and sensitivity of primer and probe sets, accurate estimation of total rDNA copy numbers of each cryptic species in environmental samples, and precise determination of rDNA copies per cell of each cryptic species are desirable.

To achieve high species-specificities of primer and probe sets, we applied the TaqMan MGB based assay targeting the LSU D8/D10 region that showed high divergence between various species of *Ostreopsis* and high homogeneity among strains of each target species [44]. The results of cross-reactivity of primer and probe sets to various strains of *Ostreopsis* and other benthic dinoflagellates showed that the target species including cryptic species are able to be specifically detected by using the primer and probe sets developed in this study. Our method proved to be extremely sensitive over a wide dynamic range with a demonstrated detection limit of about ten copies of the four species of *Ostreopsis*, suggesting that detection of fewer than 0.03 cells g^−1^ fresh weight algae of *O.* cf. *ovata* and 0.01 cells g^−1^ fresh weight algae of *Ostreopsis* sp. 1 may be possible. This level of sensitivity is probably not necessary for environmental monitoring as such low abundances pose minimal environmental risk. Our results also showed that the Ct measurements were highly reproducible for a given DNA template quantity with a dynamic range of 6 orders of magnitude (CV_Ct_<1.1%, Table S1). The data obtained from our results indicated that the qPCR method can quantify reliably and accurately the number of cells even if only one cell of the target species was included in the sample.

One of obstacles in obtaining accurate and reproducible results of total number of copies of target species for qPCR analysis of environmental samples is the presence of inhibitory compounds [12]. In order to overcome this problem, crude cell lysate and dilution methods (e.g., 1∶10–1∶100; see Galluzzi et al. [7], Kamikawa et al. [9]and Perini et al. [19]) can be used to relieve inhibition. But given that DNA extracted from environmental samples can vary in quantity and quality, dilution alone may not be adequate to overcome this problem reliably. Recently, several approaches for removing inhibitory substances associated with genomic DNA from HAB species is to use extraction buffers containing CTAB (cetyltrimethylammonium bromide) or specialized DNA extraction kits such as the DNeasy Plant Mini Kit (QIAGEN, Tokyo, Japan) and PowerSoil DNA Isolation Kit (MO Bio, California, USA) [6], [9], [10], [11], [12], [13], [15], [20], [21], [22], [23], [25]. In this study, we extracted and purified the genomic DNA from the samples by using DNeasy Plant Mini Kit in order to minimize effects of PCR inhibitory substances in the samples. Simultaneously, we monitored amplification efficiencies (AEs) of all samples tested. We showed that inhibition of PCR amplification was observed in environmental samples even when extracted and purified using a commercial kit, when circular exogenous plasmid DNA was added. In contrast, no inhibition was observed in those same environmental samples containing linearized exogenous plasmid DNA. Furthermore, the AEs of the linearized exogenous plasmid were not significantly different between the various target species of *Ostreopsis*, or compared to AEs using genomic DNA extracted from cultured cells of the *Ostreopsis* spp. or from environmental samples containing cells of *Ostreopsis* spp.

Our results also showed that the Ct value of circular plasmid was greater than that of linearized plasmid. Hou et al. [26] also showed that circular plasmid DNA had a higher Ct compared to the linear DNA. This led to overestimation of the number of copies of the target species. To overcome the problems related with circular plasmid, all standard curves were constructed using dilution series of linearized plasmid in this study. Monitoring of DNA amplification efficiencies (AEs) in all samples and use of linearized plasmid for construction of a standard curve are important for accurate and reproducible estimation of total number of copies of target HAB species in environmental samples by an absolute qPCR.

When any DNA purification treatment is used for the purpose of removal of PCR inhibitory substances, DNA recovery needs to be monitored, since the more the sample is manipulated, the greater the possibility of negatively impacting DNA recovery efficiency [12]. Coyne et al. reported that, in their relative qPCR method, RE could be monitored by addition of external reference plasmid to samples [12]. In our study, we monitored DNA recovery efficiencies of all samples by qPCR quantification of exogenous plasmid DNA. Our results indicated that qPCR quantification results using linearized plasmids and considering normalization of RE was significantly higher and was closer to the actual number of plasmid spiked into a sample than the results by qPCR without consideration of normalization. Monitoring of DNA recovery efficiencies (REs) is important for an accurate and reproducible estimation of the total number of copies of target HAB species in environmental samples by qPCR.

To develop and establish a new qPCR method for the enumeration of each HAB species including cryptic species, precise determination of number of rDNA copies per cell of the target species is essential. Previous reports have found a significant variation in number of LSU D1/D2 rDNA copies per cell determined by qPCR among the different strains of *O.* cf. *ovata* isolated from various regions of the Mediterranean Sea and a significant variation in number of rDNA copies per cell between cells cultured at different growth phases (i.e. 6^th^ and 28^th^ day) within the same strain [19]. In contrast,no significant variation in copy number per cell of *O.* cf. *ovata* determined by qPCR was found among environmental samples collected from the regions of the Mediterranean Sea [19].

In our study, no significant variation in LSU D8/D10 rDNA copy number per cell was found among strains of each species of *Ostreopsis*, except for *Ostreopsis* sp. 6, isolated from Japanese coastal waters. The reason why the CV_copy_ of *Ostreopsis* sp. 6 was high (>55%) is thought to be due to the genetic diversity between D-2 clade strains s0587 and ISC06/ISC07. No significant decrease in rDNA copy number of *Ostreopsis* sp. 1 during different growth stages was found in our study. These results and the finding that the number of rDNA copies per cell of each species of *Ostreopsis* isolated from Japanese coastal waters was not as variable as described above, encouraged us to use number of rDNA copies per cell of target species for cell enumeration of each *Ostreopsis* species in environmental samples by qPCR.

In order to obtain an accurate number of rDNA copies per cell of *Ostreopsis* species, we determined the number of LSU D8/D10 rDNA copies per cell of cultured *Ostreopsis* and from *Ostreopsis* cells isolated from environmental samples. We took this approach because the number of rDNA copies per cultured cell of Mediterranean *O.* cf. *ovata* seemed to be different from cells isolated directly from environmental samples [19]. In addition, several recent publications used cells of target species in environmental samples as a reference for their qPCR enumeration methods rather than cells cultured under ‘artificial’ laboratory conditions [12], [19]. Our comparisons indicated that although there were slightly lower numbers in rDNA copies per cell of various cultures of Japanese *Ostreopsis* species compared to *Ostreopsis* in environmental samples from Japanese coastal areas, no significant difference was found. *Ostreopsis* sp. 5, from cultures or environmental samples showed no difference in rDNA copies per cell. Therefore, cell numbers of each species in environmental samples were calculated by division of total number of target genes of each species in the samples not only by target gene copy numbers per cultured cell of each species but also by those per cell in environmental samples. When the enumeration results were compared to total microscopy counts of *Ostreopsis* spp. in the environmental samples, the numbers using rDNA copies per cell from environmental samples were closer to the microscopy counts than those from cultures. Thus, estimates of rDNA copies per cell from environmentally isolated cells are more useful for cell enumeration of target species in environmental samples by qPCR method, because the numbers may represent the ‘average’ for numbers of various strains in environmental samples. For determination of the number of rRNA copies per cell of each species, we analyzed single cells collected from environmental samples as templates by qPCR considering normalization with RE, because cryptic species such as *O.* cf. *ovata* and *Ostreopsis* sp. 1 generally co-occur in environmental samples collected from Japanese coastal areas and it was impossible to estimate abundance of each species due to the similarity of their morphological features. The CV_copy_ value of both culture and environmental sample showed high reproducibility in *O*. cf. *ovata* and *Ostreopsis* sp. 1. The data indicated 28S rDNA copy number was quantified reliably and accurately. The fact that cell enumeration results calculated with the number of rDNA copies per cell of each species were always close to the microscopy counts suggests that number of rDNA copies of each species determined by using single cells isolated from environmental samples as templates were reliable and applicable to qPCR for detection and enumeration of HAB species containing cryptic species.

The LSU rDNA copy number of *O.* cf. *ovata* from the Mediterranean Sea [19] was lower than that of Japanese *O.* cf. *ovata*. One possible reason for the difference is that genomic characteristics of the Japanese strains may be different from those isolated from the Mediterranean Sea, even if these strains are closely related as determined by phylogeny using LSU [44]. The other possible reason is that the methods for estimation of the rDNA copy numbers are different between the two studies. We estimated number of rDNA copies per cell of Japanese species by using single cells isolated from environmental samples, whereas Perini et al. [19] determined number of gene copies of *O.* cf. *ovata* from the Mediterranean Sea using ‘multiple’ cells that were collected from culture samples or environmental samples. Multiple cells collected from cultures or environmental samples may contain not only ‘healthy’ cells but also ‘ghost’ or ‘dead’ cells. In fact, single cells selected which were abnormal-shaped or less-colored sometimes showed no DNA amplification. Existence of such ‘ghost’ or ‘dead’ cells in samples may cause underestimation of number of rDNA copies per cell of target species in some cases. To circumvent the problem, we used ten healthy cells for determination of rDNA copy number of each species. Our results estimating the LSU rRNA gene copy number of Japanese *O.* cf. *ovata* may correspond with the report of Godhe et al. [47] that found the number of rDNA copies per cell for various diatoms and dinoflagellates were significantly correlated to the biovolumes of the cells (y = −0.61+1.22x, y: log biovolume µm^−3^ cell^−1^, x: number of rDNA copies per cell). The biovolume of *O.* cf. *ovata* collected from Japanese coastal areas was more than 10,000 µm^−3^ [44] and using their linear regression the number of rDNA copies per cell estimated was more than 10,000.

Distribution and co-existence of *O.* cf. *ovata* and *Ostreopsis* sp. 1 in Honshu (main island of Japan) and Shikoku Island reported by Sato et al. [44] was ascertained by qPCR results in this study. In case of distribution of *Ostreopsis* sp. 6 in Japan, this species was detected not only in Okinawa region but also in Shikoku Island by qPCR in this study, which suggests this species is distributed in the temperate regions as well as tropical regions.

In conclusion, we developed new qPCR method that is applicable for enumeration of each target HAB species in environmental samples where cryptic species co-occur. This method is expected to be a powerful tool for monitoring of various HAB species containing cryptic species and for development of a HAB forecast system in various coastal areas in the near future.

## Supporting Information

Figure S1 **28S rDNA copy number per cell during various culture stages.** Ribosomal DNA copy number of cell of *Ostreopsis* sp. 1 at early exponential growth phase, late exponential growth phase and stationary phase were determined, respectively. *Ostreopsis* sp. 1 s0716 was cultured in f/2 medium and ten cells were isolated at 6 days after subculture (early exponential growth phase), 19 days after subculture (late exponential growth phase) and 26 days after subculture (stationary growth phase). Number of ribosomal DNA copies per cell of each sample was determined by qPCR considering normalization with RE as described above. Number of 28S rDNA gene copies of each sample was determined as average number of rDNA copies of ten single cells isolated from each culture samples. Number of 28S rDNA copies in early exponential growth phase was not significantly different from that in stationary phase (*p*>0.05). Numbers on y-axis represented Log_10_ number of 28S rDNA copies per cell. Values are mean ± SD.
(TIF)

Figure S2 **Standard curves of** ***Ostreopsis*** **genomic DNA and control plasmid with DNA extracted from environmental samples.** Standard curves using species-specific primer and probe sets (Black circle) and pGEM primer and probe set (White circle) were constructed with 10-times dilution series (from 10^−2^ to 10^−6^ dilution) of environmental samples (Nos. 1–4, Table 5, Fig. 8) using average for Ct values in triplicate experiments (A: *O*. cf. *ovata*. B: *Ostreopsis* sp. 1. C: *Ostreopsis* sp. 5. D: *Ostreopsis* sp. 6). Error bars represent standard deviation of triplicate PCR reactions. Statistical analysis was performed using Student t-test between amplification efficiencies of genomic DNA of each *Ostreopsis* species and pGEM plasmid. Significance was accepted at *p*<0.05.
(TIF)

Table S1 **Reproducibility of the Q-PCR assay**
(PPT)
