# Supplementary figures and images for: Quantitative PCR Method for Enumeration of Cells of Cryptic Species of the Toxic Marine Dinoflagellate Ostreopsis spp. in Coastal Waters of Japan
Source: PLoS One. 2013 Mar 13;8(3):e57627. doi: 10.1371/journal.pone.0057627 (PMC3596365; doi:10.1371/journal.pone.0057627)

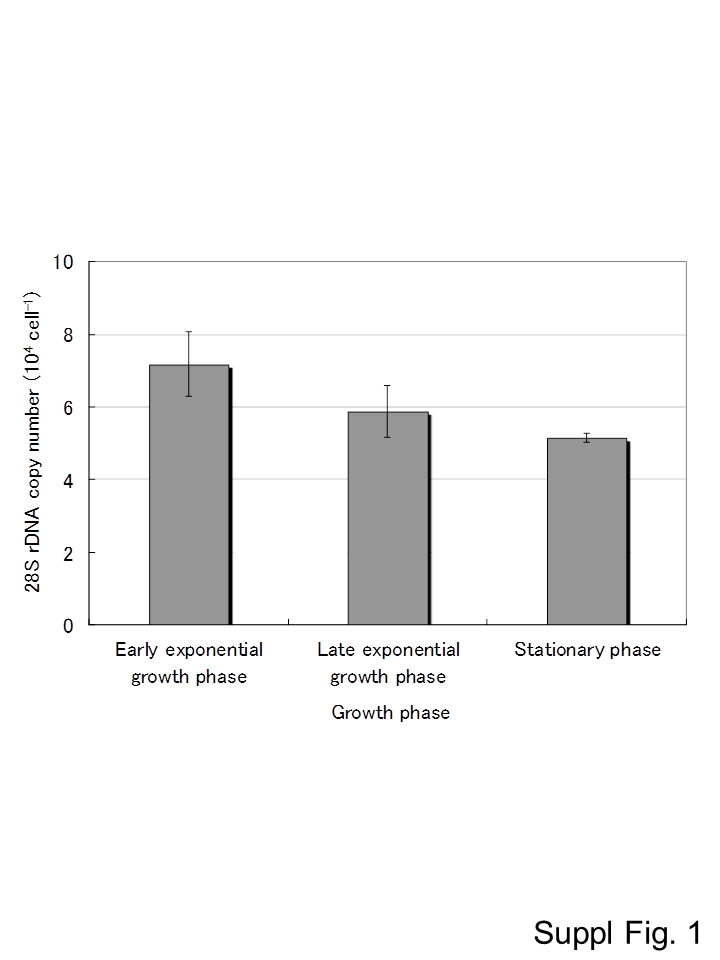

Supplement: Figure S1 — 28S rDNA copy number per cell during various culture stages. Ribosomal DNA copy number of cell of Ostreopsis sp. 1 at early exponential growth phase, late exponential growth phase and stationary phase were determined, respectively. Ostreopsis sp. 1 s0716 was cultured in f/2 medium and ten cells were isolated at 6 days after subculture (early exponential growth phase), 19 days after subculture (late exponential growth phase) and 26 days after subculture (stationary growth phase). Number of ribosomal DNA copies per cell of each sample was determined by qPCR considering normalization with RE as described above. Number of 28S rDNA gene copies of each sample was determined as average number of rDNA copies of ten single cells isolated from each culture samples. Number of 28S rDNA copies in early exponential growth phase was not significantly different from that in stationary phase (p>0.05). Numbers on y-axis represented Log10 number of 28S rDNA copies per cell. Values are mean ± SD. (TIF) [file pone.0057627.s001.tif]

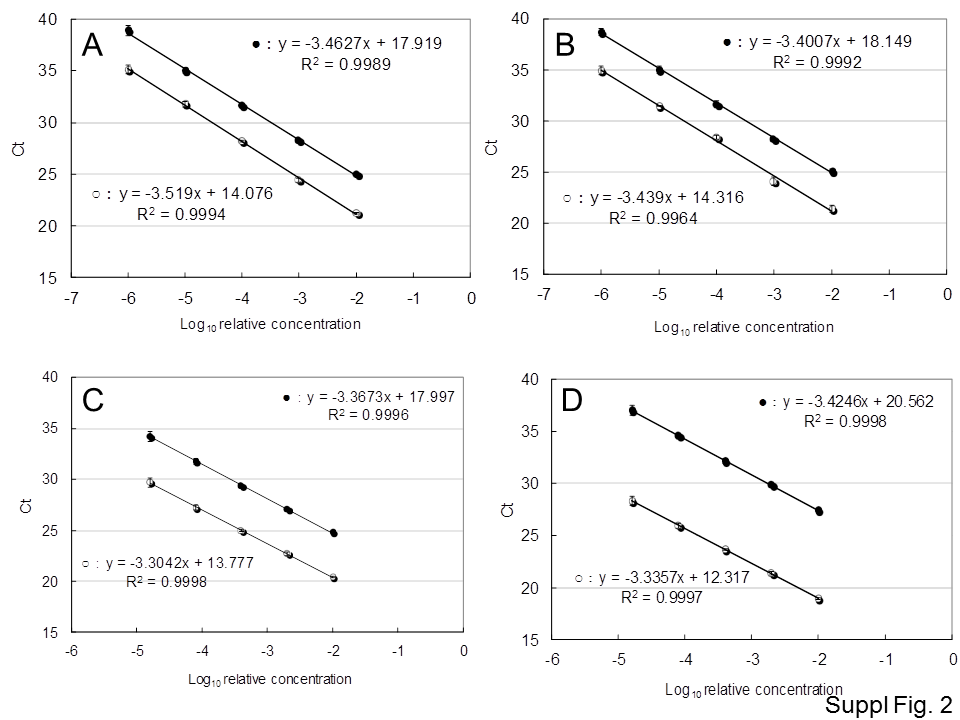

Supplement: Figure S2 — Standard curves of Ostreopsis genomic DNA and control plasmid with DNA extracted from environmental samples. Standard curves using species-specific primer and probe sets (Black circle) and pGEM primer and probe set (White circle) were constructed with 10-times dilution series (from 10−2 to 10−6 dilution) of environmental samples (Nos. 1–4, Table 5, Fig. 8) using average for Ct values in triplicate experiments (A: O. cf. ovata. B: Ostreopsis sp. 1. C: Ostreopsis sp. 5. D: Ostreopsis sp. 6). Error bars represent standard deviation of triplicate PCR reactions. Statistical analysis was performed using Student t-test between amplification efficiencies of genomic DNA of each Ostreopsis species and pGEM plasmid. Significance was accepted at p<0.05. (TIF) [file pone.0057627.s002.tif]

## Slide 1
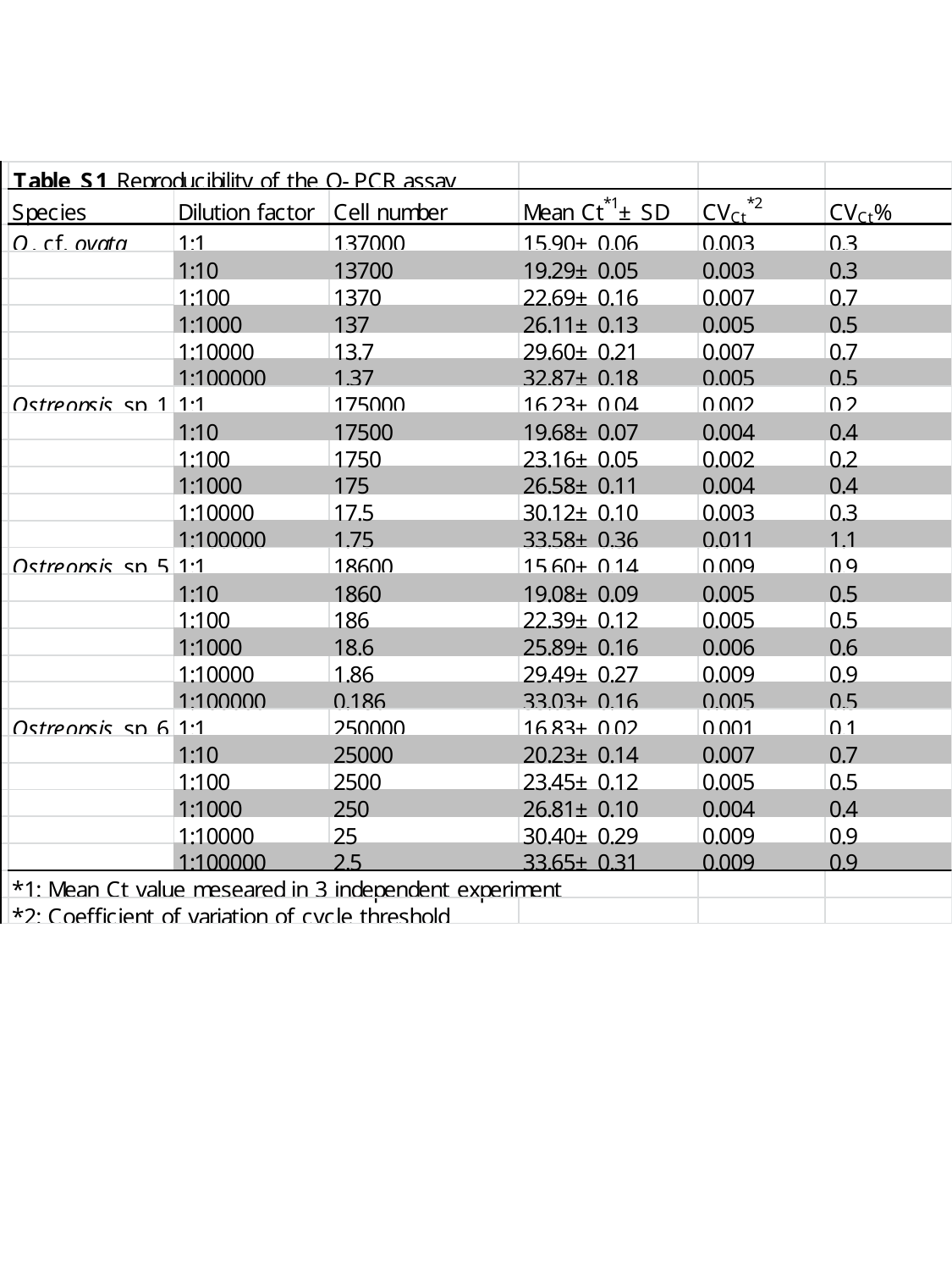

Supplement: Table S1 — Reproducibility of the Q-PCR assay (PPT) [file pone.0057627.s003.ppt]
